# Physiological and metabolomic analysis reveals maturity stage-dependent nitrogen regulation of vitamin C content in pepper fruit

**DOI:** 10.3389/fpls.2022.1049785

**Published:** 2023-01-13

**Authors:** Lu Zhang, Fen Zhang, Yuan Wang, Xiao Ma, Yuanpeng Shen, Xiaozhong Wang, Huaiyu Yang, Wei Zhang, Prakash Lakshmanan, Yuncai Hu, Jiuliang Xu, Xinping Chen, Yan Deng

**Affiliations:** ^1^ Interdisciplinary Research Center for Agriculture Green Development in Yangtze River Basin, College of Resources and Environment, Southwest University, Chongqing, China; ^2^ Key Laboratory of Low-carbon Green Agriculture in Southwestern China, Ministry of Agriculture and Rural Affairs, Southwest University, Chongqing, China; ^3^ Key Laboratory of Efficient Utilization of Soil and Fertilizer Resources, Southwest University, Chongqing, China; ^4^ Sugarcane Research Institute, Guangxi Academy Agricultural Science, Nanning, Guangxi, China; ^5^ Queensland Alliance for Agriculture and Food Innovation, University of Queensland, St. Lucia, QLD, Australia; ^6^ School of Life Sciences, Technical University of Munich, Freising, Germany; ^7^ National Academy of Agriculture Green Development, China Agricultural University, Beijing, China

**Keywords:** *Capsicum annuum* L., nitrogen level, maturity stage, ascorbic acid, dehydroascorbic acid

## Abstract

Pepper is one of the most vitamin C enriched vegetables worldwide. Although applying nitrogen (N) fertilizer is an important practice for high fruit yield in pepper production, it is still unclear how N application regulates pepper fruit vitamin C anabolism at different maturity stage. To further the understanding, we combined physiological and metabolomic analysis to investigate the fruit vitamin C content (including ascorbic acid (AsA) and dehydroascorbic acid (DHA)), related enzyme activity and non-targeted metabolites of field-grown chili pepper produced under different N levels at mature green and red stages. The results showed that increasing N application reduced AsA content in pepper fruit at both maturity stages, but highly elevated DHA content only at mature green stage. Regardless of N application level, AsA content displayed an increasing trend while DHA content was reduced as pepper fruit maturity advanced, resulting in a higher content of total vitamin C at the mature green stage. The L-galactose pathway, D-galacturonate pathway, and myo-inositol pathway were identified for AsA biosynthesis. The involved precursor metabolites were mainly negatively regulated by increasing N application, and their accumulation increased when pepper fruit developed from green to red stage. Meanwhile, the activities of key enzymes and metabolites in relation to degradation and recycling processes of AsA and DHA were increased or did not change with increasing N application, and they were differently influenced as fruit maturing. As a result, the recommended N application level (250 kg N ha^-1^) could maintain relatively high total vitamin C content in pepper fruits without yield loss at both maturity stages. These findings highlight the importance of optimizing N application level to maximize vitamin C content in pepper fruits, and provide a better understanding of the maturity stage-dependent N regulation on vitamin C anabolism.

## 1 Introduction

Vitamin C is one of the most abundant water-soluble antioxidants in plants, and it plays crucial roles in human health, such as scavenging free radicals and reducing cancer risk (Paciolla et al., 2019). Its roles as prophylactic and adjuvant medical treatment for COVID-19 have been demonstrated recently (Feyaerts and Luyten, 2020). Humans can only obtain vitamin C from the diets, in which vegetables and fruits are the main source (Mellidou and Kanellis, 2017). Pepper (*Capsicum annuum* L.) is a popular vegetable and condiment consumed worldwide, and is widely recognized with the most vitamin C enriched in fresh fruits (Wahyuni et al., 2013). Producing pepper fruits with high vitamin C content is beneficial to better human nutrition.

In plants vitamin C includes two forms, ascorbic acid (AsA) and dehydroascorbic acid (DHA), with AsA as the main active form (Marín et al., 2004). Currently, four biosynthetic pathways have been reported for AsA, i.e., L-galactose pathway (Smyrnoff-Wheeler pathway), D-galacturonate pathway, L-gulose pathway and myo-inositol pathway (Akram et al., 2017). Previous studies have indicated that many plants differ in their pathways to synthesize AsA, and may switch pathways during fruit ripening (Badejo et al., 2012; Liao et al., 2021). In an extensive review, Tyapkina et al. (2019) conclude that there is no unequivocal evidence for the existence of a predominant pathway of AsA biosynthesis in fleshy fruits. In pepper plants, the L-galactose pathway has been studied by several researchers (Alós et al., 2013; Gómez-García and Ochoa-Alejo, 2016; Rodríguez-Ruiza et al., 2017), but there is very limited understanding about the other AsA biosynthesis pathways. Then, synthesized AsA can be degraded through oxidation into monodehydroascorbic acid (MDHA) and DHA (Paciolla et al., 2019); meanwhile, MDHA can be directly reduced again into AsA while DHA can participate in the recycling pathway of ascorbate-glutathione (AsA-GSH) cycle to regenerate AsA (Gallie, 2013). DHA, if not rapidly recycled into AsA, will be irreversibly hydrolyzed to threonate, oxalate, oxalyl threonate, or tartrate (Truffault et al., 2017), representing a net loss of total vitamin C. Moreover, AsA is mobile in plants and can be transported from source organ to sink organ, such as from leaves to fruits (Chiaiese et al., 2019). As a result, vitamin C accumulation in a certain plant organ or tissue can be regulated at different steps in relation to AsA/DHA biosynthesis, degradation, recycling and translocation.

Application of nitrogen (N) fertilizer is a common practice in intensive vegetable production to increase yield. The relationship between N fertilizer level and vitamin C content has been reported frequently for different vegetables. Most results have shown that high N application would decrease vitamin C content (Hamouz et al., 2007; Albornoz, 2016; Enrique Ochoa-Velasco et al., 2016), though some other studies report either no effect or even an enhancement in vitamin C content with increased N supply (Lata, 2014). In pepper fruits, several former researches have shown a decline in AsA content with excessive N supply (Mozafar, 1993; Lu et al., 2018), but little is known about DHA content. Additionally, pepper fruits usually turn from green to red, yellow or other colors when they are fully ripe. Former studies have reported a regulatory role of maturity stage on fruit vitamin C accumulation in different pepper genotypes (Márkus et al., 1999; Sarpras et al., 2019; Cisternas-Jamet et al., 2020). However, until now it is not clear whether the effects of N application level on vitamin C content (both AsA and DHA) and the underlying regulation mechanism will change with pepper fruit development. As N nutrition is a major driver of pepper yield and quality, it is of significance to fill the research gap.

Compared with the traditional physiological methods, the development of metabolomics can help researchers to have a wide and deep overview of the changed metabolic activities for a plant as influenced by environmental conditions (Brunetti et al., 2013). Metabolite biodiversity at different fruit development stages in various plant species as well as genotypes of the same plant species have been reported (Jang et al., 2015; Sarpras et al., 2019). Recently, capsaicinoids, fatty acids and amino acids content in different parts of the fruit of the hottest Naga king chili pepper were characterized (Ananthan et al., 2018). These results indicate trade-off or parallelism or independent responses for different metabolite clusters as influenced by genetic or environmental factors, which together determines the final quality of fruits. Despite the importance of N nutrition on plant growth, a global metabolite analysis of pepper fruits at different maturity stages produced under varied N application is very lacking. Plant metabolism and its regulation are inter-connected and tightly coordinated, therefore, it is of interest to uncover the pattern of vitamin C accumulation in relation to the other metabolites change during fruit ripening under different N supply.

Given the knowledge gap of N regulation on vitamin C content in pepper fruits, the present study aims to elucidate the effect of different N application rates on vitamin C content in pepper fruits by studying related enzymes and metabolites at mature green and red stages. Here, we hypothesized that excessive N application would reduce both AsA and DHA contents, with regulation mechanism independent of maturity stage, and thus to decrease the total vitamin C content in pepper fruits. To our knowledge, this is the first detailed report on N regulation of vitamin C anabolism in pepper fruits using non-targeted metabolomic analysis.

## 2 Materials and methods

### 2.1 Field site and experimental design

A long-term field experiment was started in 2018 and the present study was carried out in 2020, the third pepper cropping season. The experimental site locates in Weituo Town, Hechuan District, Chongqing City, China (30°01′N, 106°13′E). This region has a subtropical monsoon climate, with an average annual temperature of 18.2°C and precipitation of 1150 mm. The soil is classified as alluvium, and the primary properties of 0-20 cm soil layer before setting up the experiment were as follows: pH 5.65 (soil-water ratio 1:2.5), organic matter 9.21 g kg^-1^, total N 0.50 g kg^-1^, and available phosphorus 19.51 mg kg^-1^.

As described in a former paper (Ma et al., 2022), three N application treatments were established following a completely randomized block design: N0 (control; 0 kg N ha^-1^, 140 kg P_2_O_5_ ha^-1^, 300 kg K_2_O ha^-1^), N250 (recommended N level; 250 kg N ha^-1^, 140 kg P_2_O_5_ ha^-1^, 300 kg K_2_O ha^-1^) and N400 (excessive N level used by farmers; 400 kg N ha^-1^, 290 kg P_2_O_5_ ha^-1^, 230 kg K_2_O ha^-1^). Each treatment had four replicate plots, and each plot had an area of 46.6 m^2^ (8.25 m × 5.65 m), with a 1.5-meter protection space between plots. Chili pepper variety “Xinxiang 8” was used for this experiment, and a total of 160 pepper plants (0.6 m row spacing × 0.4 m plant spacing) were grown in each plot. The chemical fertilizers used were urea (46% N), superphosphate (12% P_2_O_5_), and potassium sulphate (50% K_2_O). For each treatment fertilizers were split applied with different nutrient ratios on different dates, and the detailed application information is presented in **Table S1**. The experiment was conducted from 12 May to 6 September, 2020. Except the fertilization practice, all the other field management was done according to the local practice for pepper production.

### 2.2 Plant sampling and processing

Pepper fruits were sampled at mature green (30 July) and red (25 August) stage, respectively. For each sampling, 10 representative plants of similar growth were selected from each plot and a total of 20 fresh fruits (two fruits per plant) were sampled from the same parts of the chosen plants. Then the sampled pepper fruits were divided into two parts and processed for different parameter assay. To be specific, for each plot, half of the sampled pepper fruits were used for measurement of fruit exterior quality and N concentration as described below; the other half fruits were quickly cleaned and dissected, then stalk and seeds were removed, and the left pericarps were cut into pieces and combined into one sample. Next, homogenized subsamples of pericarp were taken and frozen immediately in liquid nitrogen. Then frozen subsamples for AsA, DHA and related enzyme activity analyses were ground to fine powder and stored at -80°C in the lab, while frozen subsamples for metabolomics analysis were quickly transported to Wuhan MetWare Biotechnology Co., Ltd., China. Overall, there were four biological replicate samples for each parameter analyzed.

### 2.3 Measurement of fruit exterior quality and N concentration

For each replicate sample, all the 10 pepper fruits were firstly measured for fruit length and single fruit fresh weight, and the average data were calculated accordingly. Then N concentration was measured for pepper pericarp. For this, all the pericarps of the 10 pepper fruits were separated, oven-dried and ground to fine powder. The N concentration was determined after digesting samples with H_2_SO_4_-H_2_O_2_ according to the Kjeldahl method (Yang et al., 2008).

### 2.4 Determination of ascorbic acid, dehydroascorbic acid, total vitamin C and related enzyme activity

The AsA and DHA contents were determined using assay kits (Suzhou Grace Biotechnology Co., Ltd, China) according to the manufacturer’s instructions. Briefly, 0.2 g ground pericarp sample was treated with 1.0 mL of pre-cooled extracting reagent and centrifuged at 12,000 rpm under 4°C for 10 min, then the supernatant was collected and treated for assay of AsA and DHA using appropriate kit. The absorbance of treated solution for AsA was then measured at 534 nm while the absorbance of treated solution for DHA was assayed at 520 nm, respectively, by using an UV-5200 spectrophotometer (Shanghai Metash Instruments Co., Ltd, China). The contents of AsA and DHA were calculated based on sample fresh weight (mg g^-1^ FW). Then the total vitamin C content was calculated as the sum of AsA and DHA contents.

Six AsA and DHA related enzymes, i.e., L-galactono-1,4-lactone dehydrogenase (GalLDH), ascorbate peroxidase (APX), ascorbate oxidase (AAO), dehydroascorbate reductase (DHAR), monodehydroascorbate reductase (MDHAR) and glutathione reductase (GR), were chosen based on former reports (Huang et al., 2014; Liu et al., 2015) and measured using assay kits according to the manufacturer’s instruction (Suzhou Grace Biotechnology Co., Ltd, China). Similarly, 0.2 g pericarp sample was extracted with 1.0 mL pre-cooled extracting solution, centrifuged at 12,000 rpm under 4°C for 15 min and the supernatant was collected. Using UV-5200 spectrophotometer (Shanghai Metash Instruments Co., Ltd, China), the absorbance of supernatant treated by corresponding kit for each enzyme was measured under different absorption wavelength, i.e., GalLDH at 550 nm, APX at 290 nm, AAO at 265 nm, DHAR at 265 nm, MDHAR at 340 nm and GR at 412 nm. To be specific, GalLDH activity was determined by the measurement of the increment rate of reduced Cyt c (L-galactolactone reduced cytochrome C), and one enzyme activity unit was defined as the production of 1 μmol reduced Cyt c per minute per gram of sample at 25°C. Activities of APX and AAO were determined by the analysis of AsA oxidation rate, and one enzyme activity unit was defined as the oxidation of 1 μmol and 1 nmol AsA per minute per gram of sample at 25°C, respectively. Activities of DHAR and MDHAR were determined by the measurement of the reduction rate of DHA and NADH, respectively, and one enzyme activity unit was defined as the production of 1 nmol AsA per minute per gram of sample at 25°C. Activity of GR was determined by the measurement of the dehydrogenation rate of glutathione (GSH), and one enzyme activity unit was defined as the oxidation of 1 μmol oxidized glutathione (GSSG) per minute per gram of sample at 25°C. Therefore, the enzyme activity was calculated and expressed as μmol min^-1^ g^-1^ FW for GalLDH, APX and GR, and as nmol min^-1^ g^-1^ FW for AAO, DHAR and MDHAR, respectively.

### 2.5 Metabolome analysis

Non-targeted metabolomics analysis was conducted for pepper pericarp samples. Each biological sample was firstly freeze-dried by vacuum freeze-dryer (Scientz-100F) and crushed to fine powder using a mixer mill (MM 400, Retsch) with a zirconia bead for 1.5 min at 30 Hz. Then 100 mg lyophilized powder per sample was extracted with 1.2 mL 70% methanol solution, vortexed 30 s every 30 min for 6 times in total, and placed in a refrigerator at 4°C overnight. Treated samples were then centrifugated at 12,000 rpm for 10 min, and the supernatant was filtrated (SCAA-104, 0.22 μm pore size; ANPEL, Shanghai, China). The global metabolites in collected supernatant samples were analyzed using an UPLC-ESI-MS/MS system (UPLC, SHIMADZU Nexera X2; MS, Applied Biosystems 4500 Q TRAP). The UPLC and mass spectrometry working conditions are described in **Methods S1**.

Qualitative and quantitative determination of metabolites was performed using the MWDB database (MetWare Biological Science and Technology Co., Ltd.). Metabolite quantification was based on the multi-reaction monitoring (MRM) mode using triple quadrupole mass spectrometry. Collision energy (CE) and de-clustering potential (DP) were optimized for each precursor-product ion (Q1-Q3) transition (Zhu et al., 2018). Metabolite content was expressed as chromatographic peak area integrals. The differentially accumulated metabolites (DAMs) between pair of N treatments in mature green and red stage fruits were determined based on the variable importance in projection (VIP) ≥ 0.8 and fold change (FC) ≥1.6 or ≤0.625 (**Table S2**). The VIP values were extracted from the OPLS-DA (orthogonal partial least squares discriminant analysis) results. A permutation test (200 iterations) was performed. Identified metabolites were annotated using the Kyoto Encyclopedia of Genes and Genomes (KEGG) compound database (http://www.kegg.jp/kegg/compound/), and then annotated metabolites were mapped to KEGG Pathway database (http://www.kegg.jp/kegg/pathway.html). Pathways with corrected *P*-values ≤ 0.05 were considered significantly enriched.

### 2.6 Statistical analysis

SPSS 25.0 (SPSS, Inc., Chicago, IL, USA) was used for the statistical analysis. The data were subjected to one-way ANOVA and significant differences between treatments were analyzed by the Duncan’s multiple range test at *P* < 0.05. The cluster heat map (normalized by Z-score) and correlation heat map (using Pearson correlation coefficients) were performed using Origin 2021 (OriginLab Corp., Northampton, MA, United States).

## 3 Results

### 3.1 External quality and N concentration of pepper fruit

Compared to no N application (N0), N250 and N400 highly increased pepper fruit length, single fruit fresh weight and pericarp N concentration at both mature green and red stages (**Table 1**). Specifically, at mature green stage, N250 and N400 lead to an increase of 18.3% and 30.7% in single fruit fresh weight, and 32.9% and 30.4% in pericarp N concentration compared with N0, respectively. More improvement by N application was observed at mature red stage, as indicated by increase of 30.8% and 33.7% in single fruit fresh weight, and 53.9% and 50.2% in pericarp N concentration under N250 and N400. However, no significant difference for these fruit parameters were detected between N250 and N400, except for higher single fruit fresh weight under N400 than N250 at mature green stage. As pepper fruits ripening, fruit length and pericarp N concentration showed increasing trend under all three N treatments, whereas single fruit fresh weight only slightly increased under N250.

**Table 1:** Fruit length, single fruit fresh weight and pericarp N concentration of pepper fruits at mature green and red stage produced under different N levels.

Growth stages	Treatment	Fruit length (cm)	Single fruit fresh weight (g)	Pericarp N concentration (g kg^-1^ DW)
Mature green stage	N0	19.90 ± 0.42 b	16.97 ± 0.42 c	8.56 ± 0.26 b
	N250	23.14 ± 0.64 a	20.08 ± 0.47 b	11.38 ± 0.10 a
	N400	24.33 ± 0.31 a	22.18 ± 0.67 a	11.16 ± 0.23 a
Mature red stage	N0	22.07 ± 0.08 b	15.91 ± 0.49 b	8.66 ± 0.10 b
	N250	25.36 ± 0.32 a	20.81 ± 0.58 a	13.32 ± 0.26 a
	N400	25.09 ± 0.40 a	21.28 ± 1.70 a	13.00 ± 0.30 a

Values (means ± SE, n = 4) followed by different letters indicate significant difference between N application levels at the same growth stage at *P* < 0.05 according to the Duncan’s multiple range test. DW, dry weight.

### 3.2 AsA, DHA and total vitamin C content in pepper fruit

**Figure 1:**
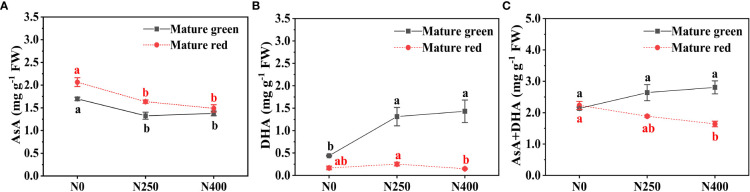
Content of AsA **(A)**, DHA **(B)** and total vitamin C (AsA+DHA) **(C)** in the pericarp of pepper fruits at mature green and red stage produced under different N levels. Values (means ± SE, *n* = 4) followed by different letters indicate significant difference between N levels at the same growth stage at *P* < 0.05 according to the Duncan’s multiple range test.

At both maturity stages, the AsA content decreased with increasing N application, but no significant difference existed between N250 and N400 (**Figure 1A**). Compared with N0, N250 and N400 lead to a decrease of 18.9% and 18.8% in AsA content at mature green stage, and 20.8% and 27.9% at mature red stage, respectively. The DHA content was highly elevated following N application at mature green stage, still with little difference between N250 and N400; whereas, at mature red stage, it went up slightly from N0 to N250, and then reduced much under N400 treatment (**Figure 1B**). Consequently, with increasing N application, total vitamin C (AsA+DHA) content in mature green fruits displayed an increasing trend, elevated by 23.5% under N250 and 31.5% under N400 in comparation to that under N0; while a reverse trend was observed in mature red fruits, as indicated by a reduction of 15.4% under N250 and 26.5% under N400 compared with N0, respectively (**Figure 1C**). Notably, AsA content increased when pepper fruits matured from green to red stage regardless of N levels, especially under N0 and N250 (**Figure 1A**). The opposite was true for DHA, with much higher levels in mature green fruits (0.44-1.43 mg g^-1^ FW) than that in mature red fruits (0.15-0.25 mg g^-1^ FW) (**Figure 1B**). As a result, as fruit maturity advancing, total vitamin C content slightly increased under no N application (N0), but significantly declined under N250 and N400 (**Figure 1C**).

### 3.3 Activity of AsA and DHA related metabolic enzymes

**Figure 2:**
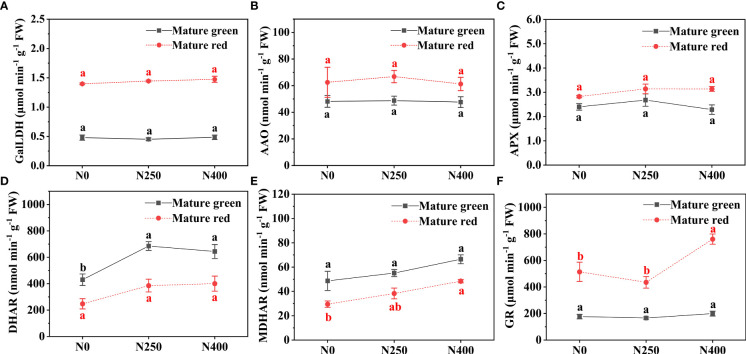
Enzyme activity of GalLDH **(A)**, AAO **(B)**, APX **(C)**, DHAR **(D)**, MDHAR **(E)** and GR **(F)** in the pericarp of pepper fruits at mature green and red stage produced under different N levels. Values (means ± SE, *n* = 4) followed by different letters indicate significant difference between N levels at the same growth stage at *P* < 0.05 according to the Duncan’s multiple range test.

The activities of GalLDH, AAO and APX were not affected by N application at both maturity stages (**Figures 2A-C**). However, N application induced DHAR and MDHAR activities, especially for DHAR at mature green stage and MDHAR at mature red stage, respectively, although no significant difference occurred between N250 and N400 (**Figures 2D, E**). To be specific, at mature green stage, N250 and N400 lead to an increase of 59.5% and 49.7% in DHAR activity compared with that under N0 (**Figure 2D**); while at mature red stage, an increasement of 29.8% and 64.1% in MDHAR activity was obtained under N250 and N400, respectively (**Figure 2E**). N application did not alter GR activity in mature green fruits, but significantly enhanced it in mature red fruits under N400 treatment (**Figure 2F**). Both DHAR and MDHAR showed much higher activity at mature green stage than that at mature red stage, whereas GalLDH, AAO, APX and GR displayed an opposite trend.

### 3.4 Differential metabolites among N treatments at two maturity stages

**Figure 3:**
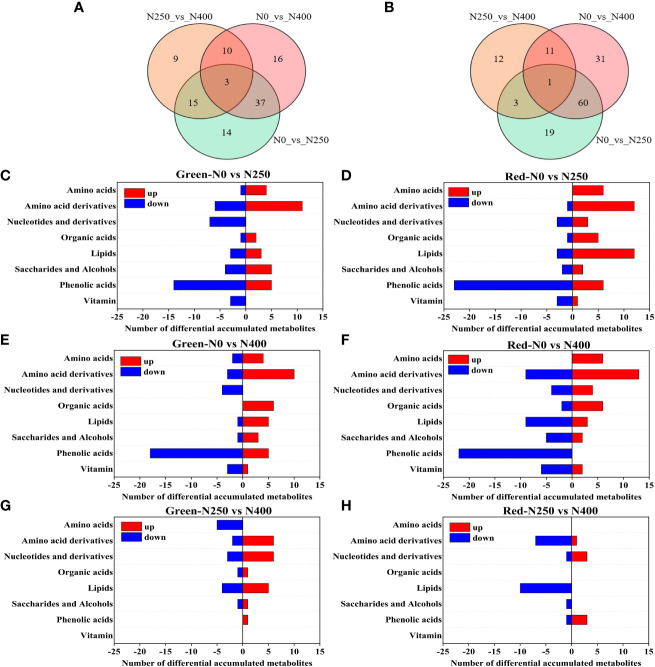
Venn diagram depicting the common and specific differential accumulated metabolites (DAMs) in the pericarp of pepper fruits between pairs of N levels at mature green **(A)** and red **(B)** stage, and the classification of DAMs in the pericarp of pepper fruits in comparison of N0 vs N250 **(C)**, N0 vs N400 **(E)** and N250 vs N400 **(G)** at mature green stage, and N0 vs N250 **(D)**, N0 vs N400 **(F)** and N250 vs N400 **(H)** at mature red stage.

In total, 388 and 411 metabolites were identified in the pericarp of pepper fruits at mature green and red stage, respectively. The OPLS-DA models were used to further compare the differences between the metabolite groups (**Figure S1**). Permutation test plot with 200 iterations showed Q^2^ values of all comparison groups exceeded 0.75 and *P*<0.005, indicating that the models were reliable (**Figure S2**). The differentially accumulated metabolites (DAMs) between pairs of N treatments were then determined based on the VIP ≥ 0.8 and FC ≥1.6 or ≤0.625 (**Table S2**). These DAMs were grouped into 7 classes, including amino acids and derivatives, phenolic acids, nucleotides and derivatives, organic acids, lipids, saccharides and alcohols, and vitamin (**Table S2**). When pairs of N treatments combined, there were a total of 104 DAMs and 3 DAMs overlapped at mature green stage (**Figure 3A**), and a total of 137 DAMs and only one shared DAM at mature red stage (**Figure 3B**). Specifically, at mature green stage, the DAMs were 69 (30 up, 39 down), 66 (34 up, 32 down) and 37 (21 up, 16 down) in N0 vs N250, N0 vs N400, N250 vs N400, respectively (**Table S2A**); while at mature red stage, the respective DAMs were 83 (47 up, 36 down), 103 (46 up, 47 down) and 23 (7 up, 16 down) (**Table S2B**). With increase of N application (N0 vs N250, N0 vs N400), at both stages the up-regulated DAMs were mainly classified into “amino acid and derivatives”, while the most down-regulated ones were “phenolic acids” (**Figures 3C-F**). At mature green stage, compared with N250, “amino acids”, slightly followed by “lipids”, were largely down-regulated while “amino acid derivatives” and “nucleotides and derivatives” were highly up-regulated under N400 treatment (**Figure 3G**); whereas, at mature red stage, the most changed metabolites in N400 treatment were down-regulated “amino acid derivatives” and “lipids”, with “lipids” followed by “amino acid derivatives” (**Figure 3H**). As a whole, much more metabolites changed at mature red stage (especially down-regulated “phenolic acids”) compared with those at mature green stage.

### 3.5 Metabolites involved in AsA biosynthesis and regeneration

**Figure 4:**
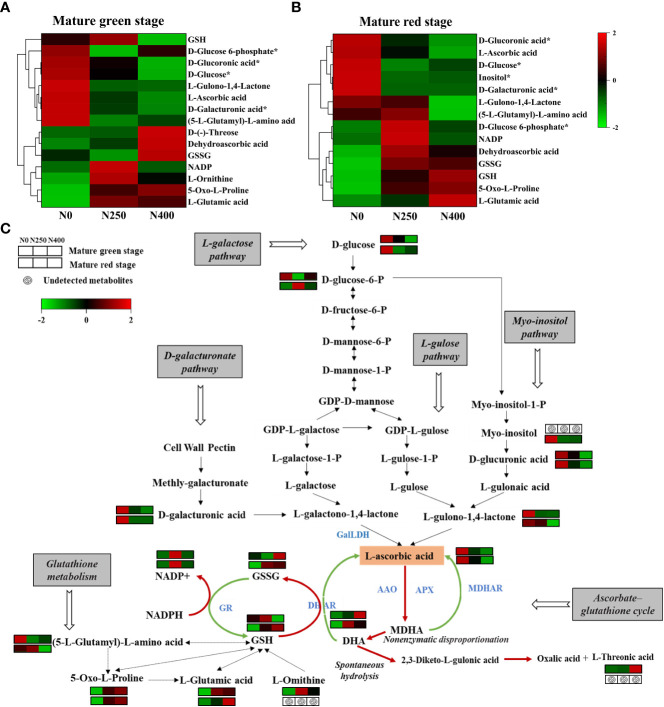
Cluster heat map showing the average accumulated metabolites (n=4) involved in AsA biosynthesis and regeneration in the pericarp of pepper fruits at mature green **(A)** and red **(B)** stage, and changes in metabolic pathways of AsA biosynthetic, AsA-GSH cycle and glutathione metabolism **(C)** as affected by N application. Relative abundance of metabolite (Z scores standardize to N (0, 1)) is shown on a red (high) to green (low) scale. The green line represents reduction reaction and the red line represents oxidation reaction in the AsA-GSH cycle, respectively.

To be specific, 15 and 14 metabolites in relation to AsA biosynthesis and regeneration were detected at mature green and red stage, respectively (**Figures 4A, B**). These metabolites were mainly associated with amino acid and derivatives, nucleotides and derivatives, organic acids, vitamin, and saccharides and alcohols (**Table S3**). In detail, at mature green stage, with N application, detected AsA synthesis precursors, i.e., D-glucose and D-glucose-6-P in the L-galactose pathway, D-galacturonic acid in the D-galacturonate pathway, D-glucuronic acid in the myo-inositol pathway, and L-gulono-1,4-lactone shared in the L-gulose pathway and myo-inositol pathway, all declined and showed remarkable parallelism with AsA (**Figure 4C** and **Table S4A**); similar trends were found at mature red stage for myo-inositol in the myo-inositol pathway and all the above precursors except for D-glucose-6-P, which showed up-regulation under N250 but down-regulation under N0 and N400 (**Figure 4C** and **Table S4B**). Among the other metabolites related to AsA-GSH cycle, L-threonic acid in the DHA hydrolysis pathway was only detected in mature green fruits, and its accumulation elevated with increasing N application, in accordance with DHA (**Figure 4C** and **Table S4A**). Meanwhile, there were no consistent accumulation patterns for most glutathione metabolism related metabolites (GSH, GSSH, (5-l-Gultamyl)-L-amino acid, L-Glutamic acid, and L-Omithine) as influenced by N treatments and fruit maturity, but NADP was consistently up-regulated by N250 while 5-Oxo-L-Proline kept increasing by N application irrespective of maturity stage (**Figure 4C** and **Table S4A, B**).

Then these DAMs were mapped to the KEGG database. Of these, 5-Oxo-L-proline, involved in glutathione metabolism (ko00480), was up-accumulated in N0 vs N250 and N0 vs N400 at both maturity stages (**Figure S3**). L-Ornithine, involved in glutathione metabolism and only detected at mature green stage, was increased in N0 vs N250 (**Figure S3A**). L-Ascorbic acid, involved in ascorbate and aldarate metabolism (ko00053) and glutathione metabolism, was decreased in N0 vs N400 at mature red stage (**Figures S3D**, **S4B**). DHA and D-(-)-threose, on the other hand, were increased in N0 vs N400 at mature green stage (**Figures S3C**, **S4A**).

### 3.6 Correlation of AsA, DHA, total vitamin C and related enzymes and metabolites

**Figure 5:**
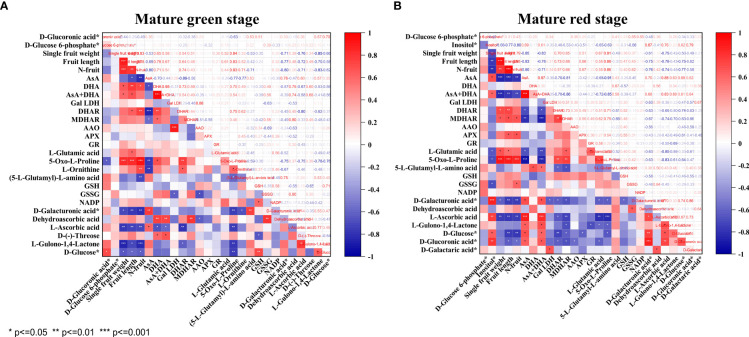
Correlation analysis of vitamin C metabolism related components, enzymes, fruit growth parameters and pericarp N concentration for pepper fruits at mature green **(A)** and red **(B)** stage. Pearson’s correlation coefficients are presented. Asterisk indicates statistically significant correlation at different level of *P* value (* *P*<0.05, ** *P*<0.01, *** *P*<0.001).

As shown in **Figure 5**, only AsA displayed consistently negative correlation with fruit pericarp N concentration (N-fruit) and 5-Oxo-L-proline at both stages of pepper fruit development (*P*<0.01). The other correlations changed as fruits turned from green to red. Specifically, AsA was highly positively correlated with D-galacturonic acid (*P*<0.01), but inversely correlated with L-ornithine (*P*<0.01), DHA (*P*<0.05), DHAR (*P*<0.001) and MDHAR (*P*<0.05) at mature green stage. At mature red stage, AsA was positively correlated with inositol, D-glucose, D-galacturonic acid, D-glucoronic acid and L-gulono-1,4-lactone (*P*<0.05), but negatively correlated with L-glutamic acid (*P*<0.05), DHAR (*P*<0.05) and MDHAR (*P*<0.001). DHA showed positive correlation with D-(-)-threose and 5-Oxo-L-proline (*P*<0.05) at mature green stage, and with (5-L-glutamyl)-L-amino acid (*P*<0.05) at mature red stage. Consequently, the total vitamin C was positively correlated with DHA *(P*<0.001) at mature green stage, but with AsA (*P*<0.001), inositol and D-glucoronic acid (*P*<0.05) at mature red stage.

The relationships between AsA and DEMs at both stages were further examined (**Table S5**). The results showed that at both stages AsA was highly negatively correlated with amino acids (i.e., 5-Oxo-L-proline, L-asparagine, L-aspartic acid, L-methionine, L-glutamine-O-glycoside, N-monomethyl-L-arginine, N-acetyl-L-methionine, L-threo-3-methylaspartate, L-glutamine-O-glycoside, S-adenosyl-L-methionine), organic acids (3-ureidopropionic acid and 2,2-dimethylsuccinic acid), saccharides (solatriose and D(+)-melezitose O-rhamnoside), free fatty acids (7S,8S-DiHODE and (9Z,12Z)-(7S,8S)-dihydroxyoctadeca-9,12-dienoic acid), while positively correlated with phenolic acids (i.e., p-coumaric acid, 1-O-salicyl-D-glucose, 4-O-glucosyl-4-hydroxybenzoic acid, 4-O-(6’-O-glucosylcaffeoylglucosyl)-4-hydroxybenzyl alcohol, salicylic acid-2-O-glucoside, 1-O-sinapoyl-D-glucose, sibiricose A3, p-coumaroylcaffeoyltartaric acid, and anisic acid-O-feruloyl glucoside).

## 4 Discussion

Pepper fruits are an excellent source of vitamin C in human diets (Wahyuni et al., 2013), and genetic or environmental factors influencing fruit vitamin C content get increasing concerns from researchers. AsA is the main biologically active form of vitamin C. Its accumulation pattern has been well characterized in many plant species, and high levels of N application is generally known to decrease AsA content (Aminifard et al., 2012; Stefaniak et al., 2020). Our results confirm this finding as a significant decrease in AsA content in pepper fruits was observed under N250 and N400 compared to N0 at both maturity stages (**Figure 1A**), resulting in a significantly negative correlation between AsA content and pericarp N concentration irrespective of fruit maturation stage (**Figure 5**). As a major component in plant antioxidant system, AsA content is usually increased when plants suffering from abiotic stress like N deficiency (Zhang X. et al., 2016). In the present study, N0 treatment caused deficient N supply for pepper plant growth and induced high AsA accumulation, while treatments N250 and N400 alleviated such stress and AsA content reduced, indicating an effective adaptation of plants to varied nutrient supply. In addition, negative effect by N application on AsA content has been explained by dilution effect through enhanced growth of plants (Lee and Kader, 2000). In this study, N application strongly promoted pepper fruit growth as indicated by the increased single fruit fresh weight (**Table 1**), which may partly account for the decreased AsA content. It is also noted that increasing N application beyond 250 kg N ha^-1^ did not further reduce AsA content, which is probably due to similar fruit N concentration and consequently no significant difference in fruit growth of plants grown under N250 and N400 treatments (**Table 1**).

In terms of metabolism, AsA content is finely regulated by its biosynthetic pathways, and previous researches have shown that different pathways may contribute to AsA accumulation during fruit ripening (Badejo et al., 2012; Zhang C. et al., 2016; Tyapkina et al., 2019). In the present work, three AsA synthesis pathways, namely L-galactose pathway, D-galacturonate pathway and myo-inositol pathway, were detected for the studied chili pepper fruits (**Figure 4C**). In a former chili pepper feeding experiment with labeled precursors, researchers found higher incorporation of labeled L-galactose into AsA than labeled myo-inositol and D-galacturonic acid, accompanied by genes involved in the L-galactose pathway being most highly expressed, and concluded a predominant role of the L-galactose pathway for AsA biosynthesis in pepper fruits and leaves (Gómez-García and Ochoa-Alejo, 2016). Besides, the L-galactose pathway has been considered as the primary route for AsA biosynthesis in immature fruits (Badejo et al., 2012), while the alternative pathway using D-galacturonic acid mainly contributes to the final maturity stage of AsA accumulation in fruit development since degradation of the cell wall is a common process during fruit ripening (Cruz-Rus et al., 2011). Here, we did not find the accumulation of L-galactose or L-galactono-1,4-lactone in the final two steps of L-galactose pathway, but the primary precursors D-glucose and D-glucose-6-phosphate were detected at both stages and showed much higher accumulation than the other precursors defined in the other two pathways (**Figure 4C** and **Table S4**). This may partly support the important role of the L-galactose pathway in AsA biosynthesis. Meanwhile, D-galacturonate in the D-galacturonate pathway were also detected at both stages, suggesting its consistent contribution to AsA synthesis during pepper fruit development. For the myo-inositol pathway, although precursor myo-inositol was only detected in mature red fruits, its following key metabolites D-glucuronic acid and L-gulono-1,4-lactone were found at both maturity stages, indicating this pathway played a part role at least at mature red stage. Further, it is noted that all the detected precursors were mostly up-regulated under N0 treatment and down-regulated by N250 and N400, in accordance with the decrease of AsA content by increasing N supply (**Figure 4C**). Thus, it can be inferred that the depress of high N supply on pepper fruit AsA content is partly due to its inhibition of the three AsA biosynthetic pathways.

As reported before, AsA content is also influenced its degradation and recycling in relation to AsA-GSH cycle (Gallie, 2013; Dell’Aglio and Mhamdi, 2020). In this study there was no significant effect of applied N on AAO and APX enzyme activity (**Figures 2B, C**), which means varied N application did not repress AsA oxidation into MDHA and DHA. However, N application greatly increased DHAR activity at mature green stage and MDHAR activity at mature red stage, respectively (**Figures 2D, E**). DHAR has been considered very important in maintaining the AsA pool and its redox state (Ding et al., 2020). Thus, the findings in this study suggest possible contribution of recycling from DHA to AsA at mature green stage, and from MDHA to AsA at mature red stage, both of which were elevated by increasing N supply. It should be mentioned that at mature green stagy, DHA content was higher under N250 and N400 than that under N0 (**Figure 1B**), which is consistent with the DHAR activity (**Figure 2D**) but inconsistent with AsA level (**Figure 1A**). Such contrary results may suggest a more important role of biosynthesis pathways than recycling in AsA pool maintenance.

Metabolites are the basis of biological phenotypes and they help us to understand biological processes and mechanisms more thoroughly (Mecha et al., 2022). In this study, more metabolites and DAMs in pepper fruits changed significantly at mature red stage compared with those at mature green stage (**Figure 3** and **Table S2**), indicating that the metabolite diversity increased as fruit maturity advanced. Specifically, amino acids derivatives and phenolic acids were more abundant at mature red stage, which means that the primary metabolisms underwent degradation-dominated processes while secondary metabolisms were stimulated as pepper fruits ripening. Correlation analysis of AsA and DEMs demonstrated that AsA was highly negatively correlated with most amino acids and organic acids, and positively correlated with phenolic acids (**Table S5**), which suggests a possible regulatory network on AsA accumulation in relation to the carbon/nitrogen balance, as partly indicated by the varied changes of metabolites involved in the glutathione metabolism (**Figure 4C**). N application may promote plant growth and increase photosynthesis at the expense of secondary metabolism by regulating carbon/nitrogen balance (Stefaniak et al., 2020). Thus, in the present study reduced pepper fruit AsA content by increasing N supply may also be partially explained by the carbon/nitrogen balance hypothesis. As nitrogen metabolism and carbon metabolism are tightly coordinated in plants (Beauvoit et al., 2018), and plant N-associated metabolism is finely regulated at transcriptional or post-transcriptional level (Zhao et al., 2015), further study is needed to uncover the regulation mechanism of AsA and the other metabolites as influenced by N nutrition.

Total vitamin C content includes accumulation of both AsA and DHA. The present results showed that while total vitamin C content was primarily determined by AsA level, DHA level at mature green stage also played a significant role (**Figure 1**). Additionally, the contents of AsA, DHA and total vitamin C changed with pepper fruit advancing maturation irrespective of N application level. Former researches have reported that AsA content would increase (Sarpras et al., 2019; Cisternas-Jamet et al., 2020) while DHA would decline (Márkus et al., 1999; Marín et al., 2004) with pepper fruits advancing maturity, and our results support these findings. It is observed that as pepper fruits developed from green into red stage, activity of GalLDH, the key enzyme in AsA biosynthesis, was highly increased in line with AsA content (**Figures 1A**, **2A**), whereas activities of DHAR and MDHAR, the two enzymes regenerating AsA from DHA and MDHA, respectively, were significantly reduced. These inverse patterns suggest that AsA biosynthesis was increased while AsA regeneration was depressed with fruit ripening. This supports the contention that AsA biosynthesis is the basis for all AsA accumulation in plants (Huang et al., 2014; Bulley and Laing, 2016). Despite higher AAO and APX activities were found at mature red stage, much lower DHA content was detected at this stage. Considering the decreased reduction of DHA into AsA by reducing DHAR activity (**Figure 2D**) and less spontaneous hydrolysis as indicated by no detection of L-threonic acid (**Figure 4C**), the low level of DHA in mature red fruits probably indicates a spatial mismatch of AsA and its oxidases (AAO and APX), which needs further study. Consequently, total vitamin C content reduced as pepper fruits ripening, with N application mainly influencing it at red stage, indicating a maturity-dependent of N regulation.

## 5 Conclusions

Producing high-yield and high-quality pepper fruits is highly valued for the economic and healthy concerns. The present study showed that compared with the recommended level of 250 kg N ha^-1^, the excessive N application used by local farmers did not further increase pepper fruit yield. Meanwhile, varied N application differently regulated total vitamin C content (AsA and DHA) of pepper fruits at mature green and red stages. Increasing N application significantly reduced AsA content at both maturity stages, while DHA content was increased at mature green stage but reduced at mature red stage. The AsA biosynthesis and AsA-GSH recycling pathways mostly explained the variation of total vitamin C accumulation, with related enzymes and metabolites differently affected by N application when pepper fruits matured. These results indicate a trade-off between pepper fruit yield and total vitamin C content by increasing N application, and suggest the need to optimize N application level for a win-win situation of high yield and high fruit quality. Although our results provide new insights into the metabolomic regulatory mechanism of different N application level on vitamin C anabolism at different pepper fruit maturity stages, there is a need to further study how and up to what extent the varied N application levels will regulate the associated genes expression and functioning underlying these metabolic processes.

## Data availability statement

The original contributions presented in the study are included in the article/**Supplementary Material**. Further requires can be directed to the corresponding authors.

## Author contributions

ZL: Conceptualization; Methodology; Data analysis; Writing-Original draft preparation and editing. CXP, DY: Supervision; Conceptualization; Writing-Original draft preparation and editing. ZF, WY, MX, SYP: Field experiment; Methodology. WXZ, YHY, ZW, LP, HYC, XJL: Writing-Reviewing and editing. All authors contributed to the article and approved the submitted version.

## Supplementary material

Supplementary Figure 1 Orthogonal partial least squares discriminant analysis (OPLS-DA) score plots for the comparisons between three N levels of the pepper fruits. **(A)** N0 vs N250, **(B)** N0 vs N400 and **(C)** N250 vs N400 are at mature green stage; **(D)** N0 vs N250, **(E)** N0 vs N400 and **(F)** N250 vs N400 are at mature red stage.

Supplementary Figure 2 Permutation test plot of OPLS-DA model with 200 iterations. **(A)** N0 vs N250, **(B)** N0 vs N400 and **(C)** N250 vs N400 are at mature green stage; **(D)** N0 vs N250, **(E)** N0 vs N400 and **(F)** N250 vs N400 are at mature red stage.

Supplementary Figure 3 The KEGG pathway map of glutathione metabolism (ko00480) in N0 vs N250 **(A, C)** and N0 vs N400 **(B, D)** at mature green and red stage.

Supplementary Figure 4 The KEGG pathway map of ascorbate and aldarate metabolism (ko00053) in N0 vs N400 at mature green **(A)** and mature red **(B)** stage.
