# Supplementary figures and images for: Physiological and metabolomic analysis reveals maturity stage-dependent nitrogen regulation of vitamin C content in pepper fruit
Source: Front Plant Sci. 2023 Jan 13;13:1049785. doi: 10.3389/fpls.2022.1049785 (PMC9880487; doi:10.3389/fpls.2022.1049785)

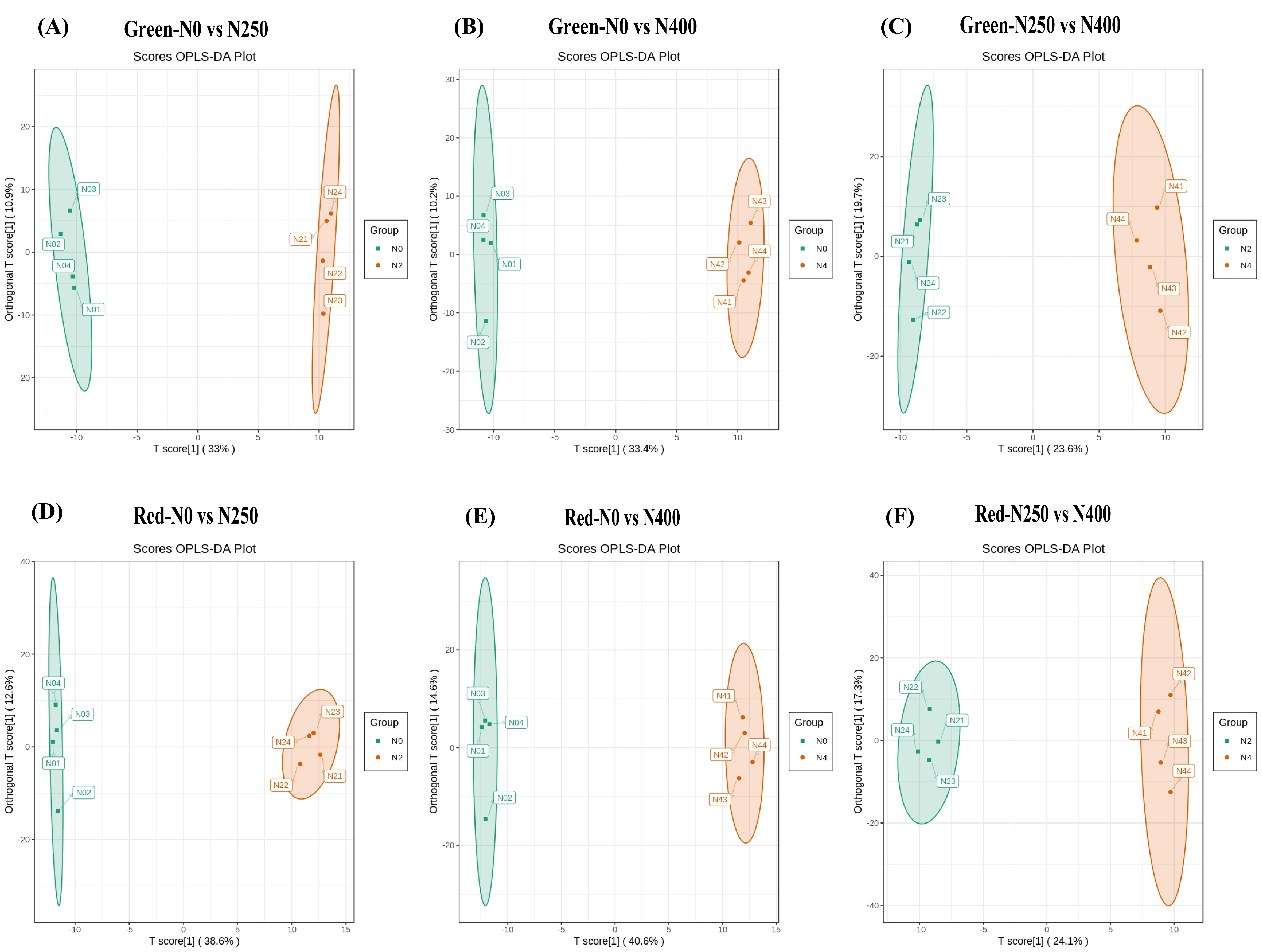

Supplement: Supplementary Figure 1 — Orthogonal partial least squares discriminant analysis (OPLS-DA) score plots for the comparisons between three N levels of the pepper fruits. (A) N0 vs N250, (B) N0 vs N400 and (C) N250 vs N400 are at mature green stage; (D) N0 vs N250, (E) N0 vs N400 and (F) N250 vs N400 are at mature red stage. [file Image_1.jpg]

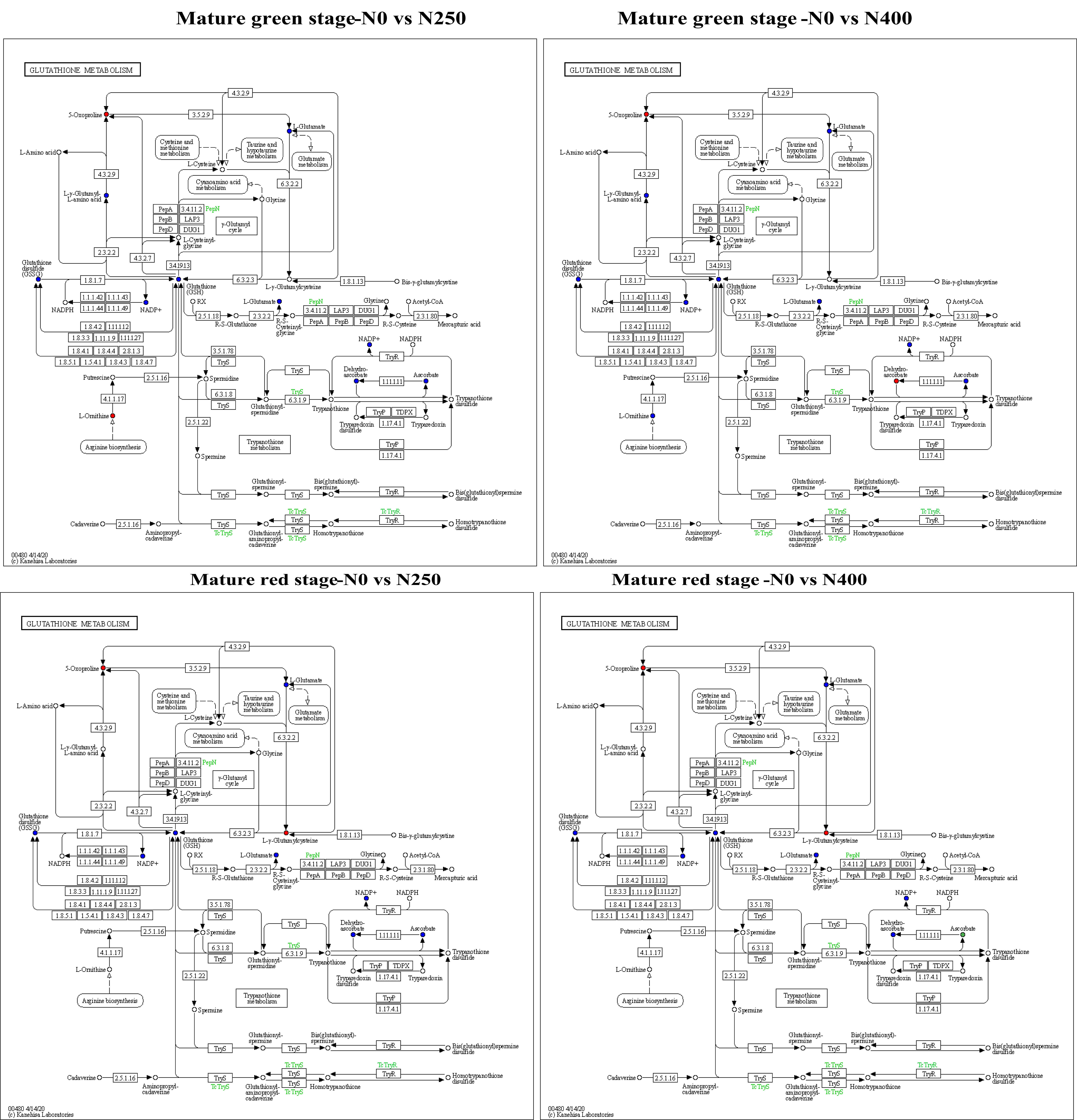

Supplement: Supplementary Figure 3 — The KEGG pathway map of glutathione metabolism (ko00480) in N0 vs N250 (A, C) and N0 vs N400 (B, D) at mature green and red stage. [file Image_3.jpeg]
